# How Significant Are Marine Invertebrate Collagens? Exploring Trends in Research and Innovation

**DOI:** 10.3390/md23010002

**Published:** 2024-12-24

**Authors:** Mariana Almeida, Tiago Silva, Runar Gjerp Solstad, Ana I. Lillebø, Ricardo Calado, Helena Vieira

**Affiliations:** 1 CESAM—Centre for Environmental and Marine Studies, Department of Environment and Planning, Campus Universitário de Santiago, University of Aveiro, 3810-193 Aveiro, Portugal; 2 3B’s Research Group, I3B’s—Research Institute on Biomaterials, Biodegradables and Biomimetics of University of Minho, Headquarters of the European Institute of Excellence on Tissue Engineering and Regenerative Medicine, AvePark—Parque de Ciência e Tecnologia, 4805-694 Guimarães, Portugal; tiago.silva@i3bs.uminho.pt; 3 ICVS/3B’s—PT Government Associate Laboratory, 4806-909 Braga/Guimarães, Portugal; 4 Nofima, Norwegian Institute of Food Fisheries and Aquaculture Research, Muninbakken 9-13, 9019 Tromsø, Norway; runar.gjerp.solstad@nofima.no; 5 ECOMARE-Laboratory for Innovation and Sustainability of Marine Biological Resources, CESAM—Centre for Environmental and Marine Studies, Department of Biology, Santiago University Campus, University of Aveiro, 3810-193 Aveiro, Portugal; lillebo@ua.pt (A.I.L.); rjcalado@ua.pt (R.C.)

**Keywords:** patents, biomaterials, application, blue biotechnology, jellyfish, sea cucumber, sponges, molluscs

## Abstract

This review is focused on the research, innovation and technological breakthroughs on marine invertebrate collagens and their applications. The findings reveal that research dates back to the 1970s, and after a period of reduced activity, interest in collagens from several marine invertebrate groups was renewed around 2008, likely driven by the increased commercial interest in these biomolecules of marine origin. Research and development are predominantly reported from China and Japan, highlighting significant research interest in cnidarians (jellyfish), echinoderms (sea cucumbers, sea urchins and starfish), molluscs (squid and cuttlefish) and sponges. Co-word analysis of the literature highlights applications in regenerative medicine, the properties of hydrolysates, and biology and biochemistry studies. Innovation and the technological landscape, however, focus on fewer taxonomic groups, possibly reflecting the challenge of sustainably sourcing raw materials, with a higher number of patents coming from Asia. Globally, jellyfish collagen is the most prominent marine invertebrate source, while Asia also emphasizes the use of collagens derived from molluscs and sea cucumbers. Europe, despite fewer patents, explores a broader range of taxonomic groups. Globally, key applications registered are mostly in medical, dental and toiletry areas, with peptide preparations spanning multiple animal groups. The food domain is notably relevant for molluscs and sea cucumbers. Market trends show a strong presence of cosmetic and supplement products, aligning with market reports that predict a growing demand for marine collagens in cosmetics and personalized nutrition, particularly in targeted health supplements.

## 1. Introduction

Collagen is a key component of the extracellular matrix of animals, providing structural and mechanical support to tissues, playing a role in biomineralization and influencing cell properties, such as adhesion, migration, proliferation and differentiation [1]. In the human body, collagen accounts for about 30% of total protein [2]. This molecule has a unique triple-helical structure made of three α polypeptide chains, which can be identical (homotrimeric) or distinct (heterotrimeric), coiled around each other. The α chain’s primary structure consists of repeating Gly-Xaa-Yaa triplets, typically glycine, proline and hydroxyproline, with the latter being a hallmark of collagens being used to identify (and quantify) its presence in biological samples. During collagen biosynthesis, key post-translational modifications (PTMs) include hydroxylation of proline residues, which stabilizes the helix and contributes to thermal stability [3], and hydroxylation of specific lysine residues, which influences fibrillogenesis, cross-linking and matrix mineralization [4]. Glycosylation is less studied but appears to be essential for collagen integrity [5]. These PTMs add a layer of information in addition to the amino acid code, resulting in diverse collagen forms [6].

To date, of the 28 collagen types described, fibrillar collagens types I–III are the most abundant in mammals and widely used in biomedical, cosmetic, and nutraceutical fields, primarily sourced from porcine and bovine skin and bones [7]. Another important non-fibrillar collagen, type IV, is the main component of the basement membrane, providing structural support and influencing cell behaviour, while fibril-associated collagens (FACIT) have interruptions within the triple-helix domain and associate with other collagen fibrils [8].

Collagens from marine organisms, including fish and invertebrates, can be isolated from their by- or co-products to produce new value-added compounds and materials, with this biobased approach being a high target on the EU policymaking agenda [9]. This goal has improved the research on the properties and applications of collagen from fish tissues [10], revealing them as safe sources with a variety of applications in human well-being and health sectors. Additionally, the study of invertebrate collagen has been driven not only by its potential applications but also by its significance in understanding animal evolution [1]. These studies have focused on the study of novel source species [11,12], improvements in isolation procedures (yields, reduction in processing time and amount of chemicals used) [13,14], life-cycle assessment [15], processing technologies and advancing their use in tissue engineering [16], wound healing [17], and drug delivery systems [18], as well as expanding the use in functional foods and nutraceuticals through the discovery of bioactive peptides [19].

**Figure 1:**
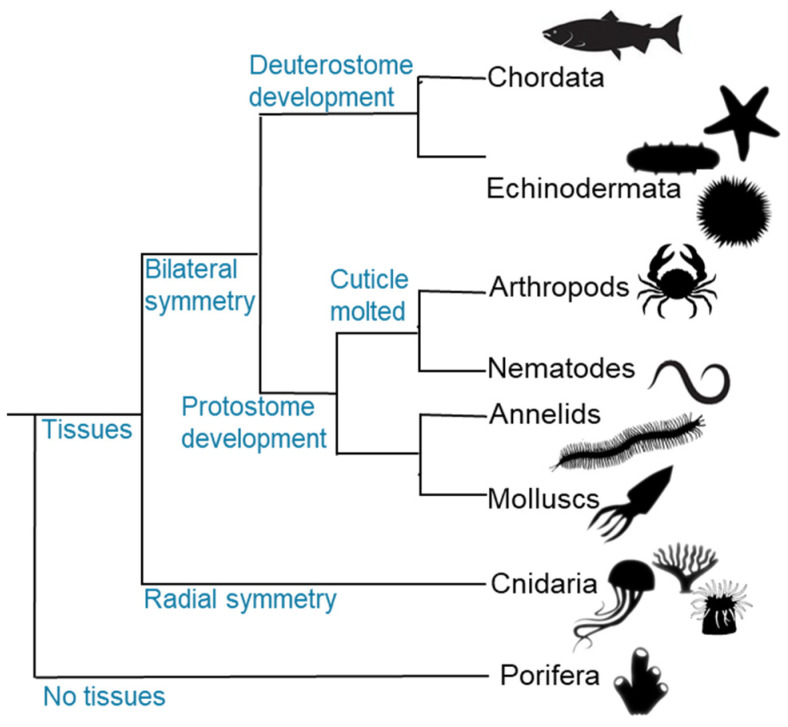
Schematic representation of a phylogenetic tree showing relationships of selected phyla analysed in the present study.

Interest in collagen-based extracellular matrices from marine invertebrates emerged early given their association to the origin of animal multicellularity and tissue evolution [8,20] and, therefore, earlier works on these collagens are likely to hold valuable insights that are worth a review (Figure 1). In this respect, this review aims to provide: (i) an overview of the physicochemical fundamentals of collagens derived from marine invertebrates; (ii) an analysis of publications and patents to provide insights into the current research and innovation landscape; and (iii) discuss potential future directions in marine collagens originating from invertebrates. This study begins with the foundational concepts of marine invertebrate collagen, establishing the relevance of publications and patent analysis. This combined approach offers a clearer understanding of the current state of the art and the evolving trends in marine collagen innovation.

### 1.1. Background on Collagen Fundamentals of Porifera—Spongin and Collagen Diversity

Sponges are ancient sessile animals consisting of a jelly-like mesohyl enclosed by two layers of cells and bodies with pores and channels for water circulation. The phylum is divided into four classes based on the skeleton composition, from which Demospongiae is the most collagen-rich one. Sponge skeletons incorporate collagen as spongins, from dispersed thin fibrils to organized macroscopic fibrous frameworks, being commonly described in Keratosa sponge species.

In sponges, collagen isolation is hindered by mineralization and insolubility in acid solutions, which generally also affects their biochemical analysis [21] and, therefore, a variety of methods allow for the discovery of several collagens’ forms present in sponges [22]. Collagen is commonly obtained from sponges via the disaggregation of the body wall using a solution containing a chaotropic agent, with the protein being generated in the fibril form [22,23].

Structural or biochemical analysis has identified fibrillar collagens, in the form of bundles, formed by the association of several hundred collagen fibrils, in diverse demosponge taxa, such as *Geodia*, *Axinella*, *Chondrosia*, *Chondrilla* and *Suberites* [12,22,24,25,26]. In glass sponges (class Hexactinellida), highly hydroxylated fibrillar collagen has been recently described in their spicules, which can act as a template for the biosilicification process [21]. A unique collagen form, spongin has been reported in certain demosponges [27]. Although its properties are not fully understood, spongin is notable for having less glycine (about 14%) compared to collagen (between 25% and 33%) and for its diverse halogen content [21].

**Figure 2:**
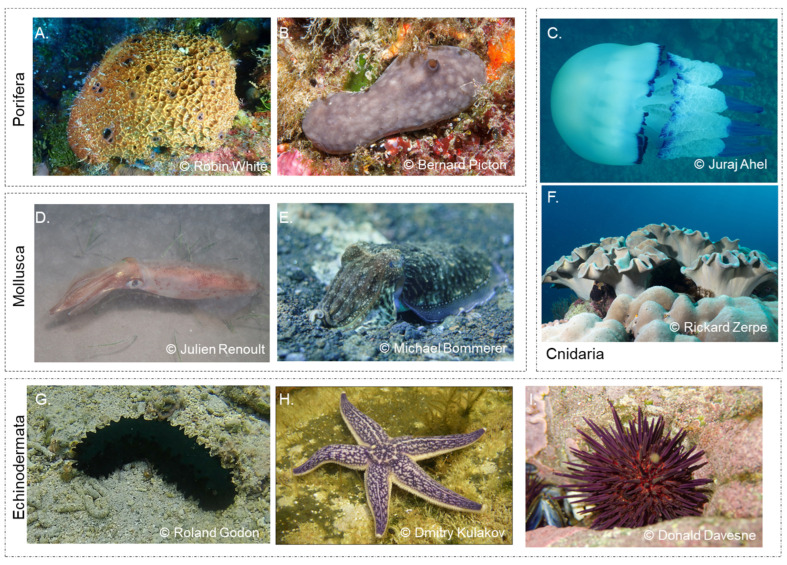
Representative marine invertebrate animals (genus or species level) from the phylum studied. Porifera ((**A**): *Ircinia*; (**B**): *Chondrosia reniformis*). Cnidaria ((**C**): jellyfish *Rhizostoma pulmo*; (**F**): coral *Sarcophyton*). Mollusca: ((**D**): squid *Loligo;* (**E**): cuttlefish *Sepia*). Echinodermata: (**G**): sea-cucumber *Stichopus*; (**H**): starfish *Asterias amurensis*; (**I**): sea-urchin *Paracentrotus lividus*. Images sourced from iNaturalist contributors under CC BY 4.0. Modifications include cropping and resizing. For license details, see https://creativecommons.org/licenses/by/4.0/ (accessed on 27 November 2024).

In family Irciinidae (Figure 2A), a collagen-like material made of filaments with globular ends and linked to carbohydrates is found within spongin matrices, forming strong yet flexible structures [28,29]. Moreover, fibrillar and non-fibrillar collagen extracted along with the filaments of *Ircinia fusca* revealed specificity for anti-collagen type I and IV human antibodies [28]. Both collagen types have also been identified in collagen fibrils in *C. reniformis* (Figure 2B). In fact, in the Mediterranean Sea, this species has been studied for several decades given its high collagen content, lack of spicules, high bioactivity and capability to modulate its mechanical properties by acting on the collagen cross-link, calling attention from the biotechnological and biomedical fields [30,31]. Numerous studies have focused on optimizing *C. reniformis* collagen extraction, biomass production and development of biomaterials for cosmetic, pharmaceutical and biomedical research [12,32,33,34].

### 1.2. Background on Collagen Fundamentals of Cnidaria—Mesoglea Collagens

Cnidaria is phylogenetically more complex than sponges and less complex than bilaterians. Their bodies consist of mesoglea, a jelly-like substance, located between two layers of cells, and their most distinguishing feature is the occurrence of a specialized cell used mainly for prey capturing and defence called cnidocyte [35]. Collagen descriptions from this phylum have been obtained from species belonging to three Cnidaria classes: Hydrozoa (includes both colonial swimmers and sessile members, such as the freshwater invertebrate model *Hydra* sp.), sessile Anthozoa (e.g., sea anemones, corals and sea pens) and swimming Scyphozoa (e.g., jellyfish) (Figure 2C). In Hydrozoa, Collagen IV has been reported in the interstitial matrix of *Hydra* in association with a fibrous collagen [36]. In Anthozoa, early studies described homotrimeric collagen molecules in the sea anemones *Actinia equina* and *Metridium dianthus* [37,38,39], while in the sea-pen *Veretillum cynomorium,* heterotrimeric collagen molecules were reported [40]. Recent works have isolated collagen-associated glycoproteins from the soft corals *Lobophytum crassum* and *Sinularia polydactyla* [41,42] and identified long and hyper-elastic collagen fibres in *Sarcophyton ehrenbergi* (Figure 2F) [41].

Research on Scyphozoa collagens has significantly expanded since initial studies on their chain structure [43]. Electrophoretic patterns showed varying subunit compositions among species. For example, collagen from the jellyfish *Catostylus mosaicus* resembles mammal collagen type I [44], while others, such as from the jellyfish *Rhopilema esculentum*, are designated as analogous to vertebrate collagen type II [45]

Other works found similarities with mammal collagens, reporting collagens characterized by two α chains, α1 and α2 in *Rhopilema asamushi* [46], (α1)3 chains in *Cyanea nozakii* [47] and α1, α2, and α3 chains in *Acromitus hardenbergi* [43,48,49]. This great variability among several jellyfish species is attributed to species-specific variations in amino acid composition and collagen molecular weight [50]. The identification of shared features between jellyfish collagens and different human collagen types suggests that jellyfish collagen may represent an ancestral form, potentially being a precursor of the structural proteins found in higher animals [51]. Indeed, the collagen produced by the company Jellagen was categorized as “collagen type 0” to highlight these similarities and ancient origin (https://jellagen.co.uk/collagen-type-0/, accessed on 4 November 2024). In addition, the higher occurrence in jellyfish blooms, caused by global warming and overfishing, among others, raises concern regarding the potential negative effects [52] but may also offer an opportunity for sustainable collagen production from abundant biomass while tackling the environmental challenge.

Specific collagens with improved mechanical properties, designed as mini-collagens, are reported for this phylum. Mini-collagens are found in the inner layer of nematocyst walls and are the smallest collagens known, with around 60 kDa molecular weight and with 12–16 Gly-X-Y repeats flanked at both ends by a polyproline stretch and a conserved cysteine-rich domain, proposed to function in collagen assembly [53]. The unique arrangement of mini-collagen trimers into disulfide-linked protofilaments provides exceptional tensile strength, while also allowing for the necessary elasticity during the rapid discharge of nematocysts [54]. These biomolecules have also been highlighted for their biomimetic potential [54].

### 1.3. Background on Collagen Fundamentals of Nematodes and Annelids—Cuticular Collagens

Cuticular collagens are reported for worm-like animals, such as nematodes and annelids, and have distinctive chemical and physical properties from fibrillar collagens. Compared to vertebrates, they are most similar to the fibril-associated collagens with interrupted triple helices [1].

The cuticle of these organisms is known to be multilayered, flexible and exoskeleton synthesized by underlying hypodermal cells that enclose the animal. While cuticles are found in several marine taxa, in nematodes and several worms, these have been described as collagen-rich [55]. Cuticle collagens from nematodes have a characteristic structure of short, interrupted blocks of Gly-X-Y sequences flanked by conserved cysteine residues [56] and cross-linked by nonreducible covalent bonds that involve tyrosine residues [55].

Annelid cuticle collagens are different from those of nematodes, as these are characterized by globular domains and a very long triple helix, high molecular masses and amino acid compositions, with about one-third of glycine being almost the only common feature [57]. An interesting example, which is species-specific, is found in the hydrothermal vent polychaete *Riftia pachyptila*, in which cuticular collagen stability is associated with a large amount of glycosylated threonine residues [58]. It is worth highlighting that while cuticular structures have also been found in some sponges, their molecular composition is yet to be determined [21]

### 1.4. Background on Collagen Fundamentals of Molluscs and Crustaceans—Collagens from Muscle Tissues

The major collagen of crustacean muscles described to date is a type V-like homotrimer observed in the prawns *Pennaeus japonicus* and *P. indicus* [59] and in the crab *Scylla serrata* [60]. In the latter, collagen presented differences within the animal body parts; leg muscles featured a higher degree of cross-linking, a higher bound of carbohydrates and increased lysine hydroxylation and reduced glycine content when compared to the collagen found in abdominal muscles [60]. These differences were associated with the functional requirements of leg muscles for locomotion and predation, while abdominal muscles contribute to normal growth and development [60].

A type V-like heterotrimer was also found in the cuttlefish *Sepia officinalis* cartilage and cornea and was associated with vertebrate cartilage minor collagens, type V and XI [61].

Collagens in molluscs have been more extensively characterized in commercial cephalopods (Figure 2D,E). Collagen extracted from the skin of squid and cuttlefish species (e.g., *Doryteuthis singhalensis*, *Dosidicus gigas*, *Kondakovia longimana*, *Illex argentinus* and *Sepia pharaonis*) was characterized as type I collagen [62,63,64,65], while in the squids *Loligo formosana* and *Todarodes pacificus*, two genetically distinct collagen types were described in the skin and/or mantle, presenting similarities to type I and V collagens, and found to possess heterotrimeric chain compositions (α1)2 α2, with the latter in a smaller amount [65,66,67].

In addition to a few works on the nutritional characterization of commercially important bivalve molluscs for human consumption [68], collagen studies have been carried out in the oysters *Crassostrea gigas* and *Pinctada martensi* [69,70]. These works demonstrated high insoluble collagens that were able to be solubilized after treatment disaggregation of collagen fibrils and revealed a similar composition as described for cephalopods: a heterotrimer structure (α1)2 α2.

One specificity within bivalves is the occurrence of collagen-like filaments formed as byssal threads [71]. Indeed, the main components of byssal threads include a set of various collagen-like structural proteins (preCols) consisting of a collagenous core sequence and two flanking domains, composed of either a soft (elastin-like), a stiff (silk fibroin-like) or an intermediate (amorphous polyglycine) chain globular domain [71,72].

### 1.5. Background on Collagen Fundamentals of Echinoderms—Mutable Collagen Tissues

Echinoderms are strictly marine animals that feature an endoskeleton composed of unique calcareous ossicles forming a framework that supports their body, and a complex system of channels and reservoirs, the water vascular system, used for movement, respiration, and feeding [35]. The phylum is divided into five classes: Crinoidea (e.g., sea-lilies and feather stars), Ophiuroidea (e.g., brittle stars), Holothuroidea (e.g., sea cucumbers), Echinoidea (e.g., sea urchins), and Asteroidea (e.g., starfish). Among these, collagen has been mostly studied in starfish, sea urchins and sea cucumbers.

One characteristic of most echinoderms across the five classes is a specific connective tissue, the mutable collagenous tissue (MCT) [73]. This collagen differs from others due to its ability to switch between rigid and flexible states, allowing the organism to adapt its body structure for different functions, for movement, defence and regeneration; this mechanism features a neural control and is only possible due to the release of specialized effector proteins [73]. At the molecular level, the collagen of sea cucumber is different from heterotrimeric vertebrate type I fibrillar collagen, consisting of homotrimers with three α1 polypeptide chains [74]. This collagen, particularly from sea cucumbers, has inspired biomimetic designs through the development of artificial materials and devices in fields, such as tissue engineering, soft robotics, and biomedical devices [75]. Although the mutability of echinoderm connective tissues is a unique characteristic of this phylum, there are also a few other examples of “mechanical adaptable tissues” among invertebrates [76].

Sea cucumbers (Figure 2G) have attracted scientific interest due to the abundance of collagen in their body walls [74]. Different species of edible sea cucumbers have been studied for their collagen content, such as *Parastichopus californicus* and *Apostichopus japonicus* from the Northwest Pacific; *Stichopus vastus*, *S. monotuberculatus* and *Holothuria parva* from the Indo-Pacific; *Cucumaria frondosa* from the Northern Atlantic and the Arctic; as well as *Australostichopus mollis* from the South Pacific [74,77,78,79,80]. In these studies, collagen extracted from sea cucumbers has primarily been identified as type I collagen, the most common type of this biomolecule found in vertebrates and the one being most investigated for potential applications [74].

Studies on collagen from the starfish *Asterias rubens* (Atlantic) and *A. amurensis* (North Pacific), which are known to significantly impact aquaculture production, are currently being performed to explore the properties of this biomolecule (Figure 2H). Indeed, the collagen of these animals has already been reported to display a structure related to that of mammalian type I [81,82].

Earlier studies on sea urchin tissues have indicated the presence of diverse and potentially specialized types of collagens in echinoids, suggesting that sea urchins may have evolved a variety of collagen structures tailored for specific functions in different parts of their bodies [83]. Despite this diversity, research also suggests a strong similarity between sea urchin collagen and that of mammalian collagen, particularly type I [84]. For instance, collagen extracted from sea urchin soft tissues, such as the peristomial membrane (the tissue surrounding the mouth) of edible species, such as *Paracentrotus lividus* and *Sphaerechinus granularis*, is primarily composed of fibrillar structures (Figure 2I) [85]. Previous studies suggest that this collagen closely resembles that of mammalian type I, exhibiting impressive mechanical properties, including high stiffness and resistance, thus making it suitable for applications in regenerative medicine and biomaterial development [86,87,88].

## 2. Results

### 2.1. Temporal Growth, Geographic Distribution and Trends in Research Topics

**Figure 3:**
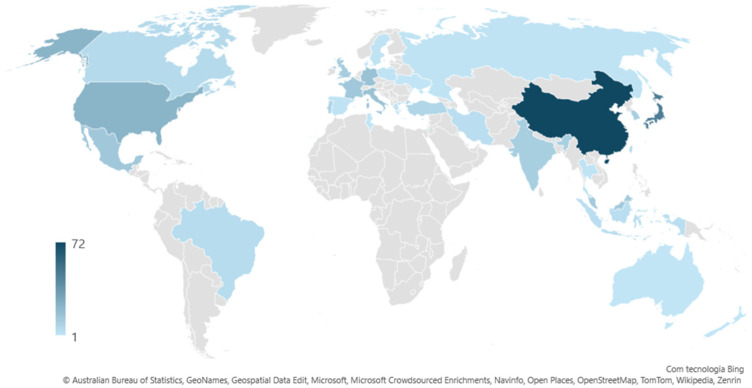
Geographical distribution of scientific publications addressing collagens in marine invertebrates.

**Figure 4:**
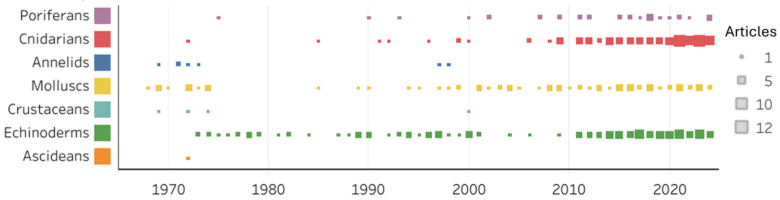
Distribution of scientific publications by year and taxonomic group addressing collagens in marine invertebrates.

The literature search on marine invertebrate collagens yielded a total of 424 publications, of which 323 publications matched the designed search criteria and were selected for deeper analysis. The highest number of selected publications originated from China and Japan (72 and 48, respectively), with significant contributions also coming from the United States of America (23), Italy (17), Germany (17), France and Mexico (14 each) and the United Kingdom (12) (Figure 3). The most studied taxonomic groups pertaining collagens from marine invertebrates were cnidarians (jellyfish: 89; corals: 5), echinoderms (sea cucumbers: 63; sea urchins: 43; starfish: 10) and molluscs (squids: 57; cuttlefish: 8), followed by marine sponges (31). The analysis of publications over time shows that research on marine invertebrate collagens increased in recent years, with over 10 publications per year being authored from the 2010s onwards, except for 2013 (Figure 4), with an increase in studies focusing on sponges and jellyfish.

**Figure 5:**
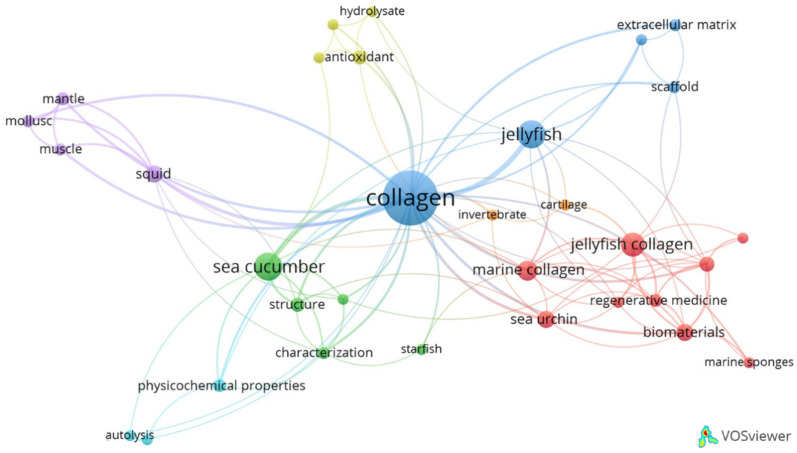
Bibliometric network visualization of the keywords associated to the scientific publications related to marine invertebrate collagen, generated using VOSviewer. Each node represents a keyword, with node size indicating the frequency of occurrence in the analysed literature. The colours correspond to clusters of closely related keywords, highlighting main areas of research. For example, “jellyfish collagen” is clustered with keywords such as “regenerative medicine” and “biomaterials”, reflecting its relevance in these fields, while “sea cucumber” is associated with “structure” and “physicochemical properties” reflecting its connection with these more fundamental studies. The edges (connecting lines) indicate co-occurrences of keywords, with the thickness of the edges representing the strength of the association.

Figure 5 presents a co-word analysis that allows for a visualization of the relationships between marine sources and various keywords found in publications addressing collagens from marine invertebrates. This analysis reveals seven distinct clusters of related terms. The blue cluster represents research focused on jellyfish collagen, with the terms “scaffold” and “extracellular matrix” suggesting an emphasis on tissue engineering and the use of this collagen to serve as a structural component in medical applications. The red cluster, although closely related to the blue cluster, focuses more broadly on various sources of marine invertebrate collagen (e.g., originating from jellyfish, sea urchins, and sponges) as a biomaterial, with an emphasis on “regenerative medicine” and “biomaterials” suggesting a strong interest in both technological and medical uses of these biomolecules. The green and emerald clusters are mostly related to the study of collagens from echinoderms, particularly sea cucumbers and starfish, and their structural and physicochemical properties, most likely related to more fundamental marine biology and biochemistry studies. The identification of mutable collagenous tissues in animals from this phylum and the efforts to explain the phenomenon of collagen aggregation and disaggregation are probably related to this. The purple and orange clusters are associated with studies addressing collagens from molluscs and, yet again, more focused on structural and physicochemical studies. Finally, the light-green cluster accounts for studies addressing the functional properties of collagen hydrolysates, namely those specifically related to sea cucumbers but, also, most likely, involving other marine invertebrate sources in general, exploring the antioxidant activity that these collagen hydrolysates display. The collagen hydrolysates, in general, have been particularly studied as potential components of nutraceutical and cosmetic formulations [89], given their higher absorption and easier metabolism, and this suggested association of hydrolysates and biological activity is certainly reflecting that.

### 2.2. Technological Development Trends, Sources, Application Areas and Demography

A total of 228 patent applications were surveyed in the present study. The first application dates back to 1997 and, in the following years, the number of patents published was relatively low (<2 per year). From 2007 onwards, the number of patent publications increased in a pronounced way, rising from 7 in 2007 to 24 in 2023, with some oscillations in numbers between those years. In 2024, there were a total of 14 patent publications.

**Figure 6:**
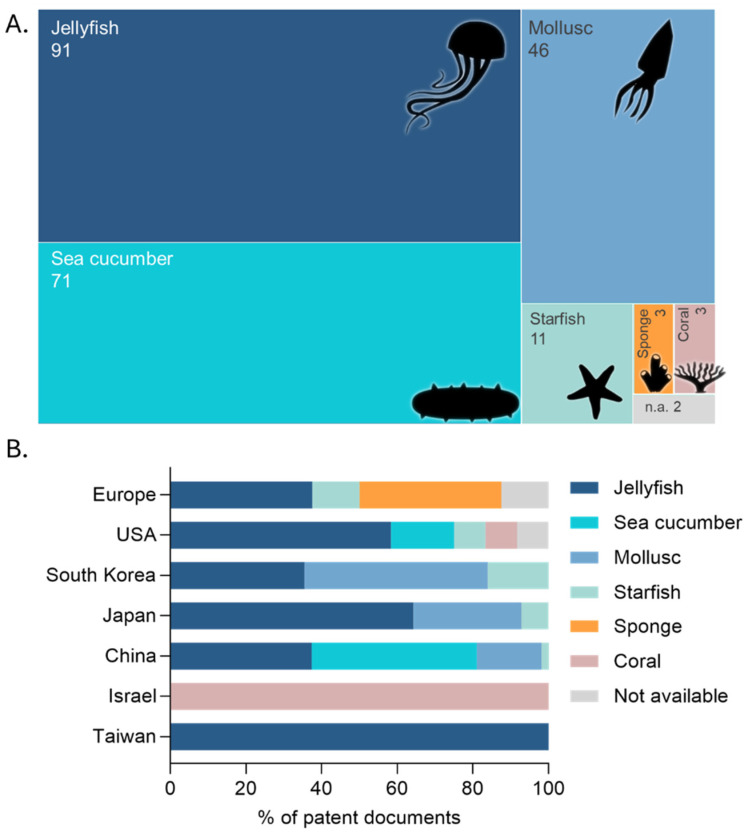
Distribution of patent publications addressing collagens by taxonomic group of marine invertebrates (**A**) and jurisdiction (**B**).

Jellyfish emerges as the most relevant source of collagen among the marine invertebrates listed with a total of 91 patent documents (Figure 6A). Sea cucumbers, with 71 patent applications and molluscs, including shellfish, squid and cuttlefish, were recorded in 46 applications. Collagens derived from starfish, sponges and corals (11, 3 and 3, respectively) show relatively low numbers of patent applications. Asia, particularly China, is the leader in the marine invertebrate collagen patent space, showing extensive activity across multiple marine invertebrate taxa, yielding 158 applications. South Korea and Japan also significantly contribute to this Asian-dominated landscape, but to a lesser extent (with 31 and 14 patents, respectively). The United States of America follows with 12 patents and Europe, including the United Kingdom, Portugal, Germany, Russia and Italy, each with a total of 8 patents. Other countries, such as Taiwan and Israel, feature two patents each (Figure 6B). Although patentability may experience different regulations across the globe, the observed predominance of Asian countries, in line with the abovementioned reports for scientific publications, is possibly related to easier access to raw materials, with China having the world’s highest aquatic food production [90].

Collagen from jellyfish is found across all countries working on this topic, while collagen from sea cucumbers and from molluscs (e.g., squid, cuttlefish, and shellfish) appears to be much more thoroughly addressed in Asia. Europe shows a more diversified but smaller-scale activity, with a notable focus on sponges and some interest in jellyfish and coral, while North America focuses primarily on jellyfish collagen, directly competing with Asian players.

**Figure 7:**
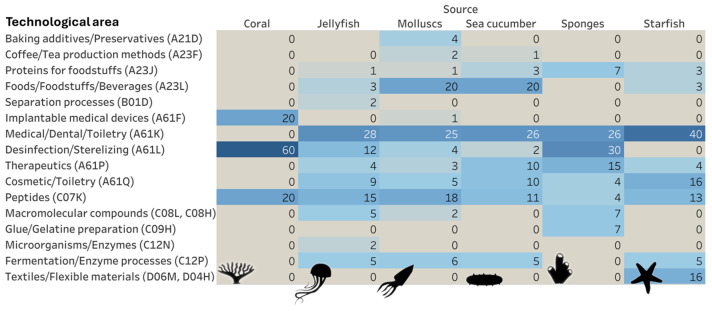
Distribution of patent publications addressing the uses of collagens by technological areas.

A comparison of the more relevant technological areas across the main animal groups is shown in Figure 7. Preparations for medical, dental or toiletry purposes (A61K) were highly relevant across all sources (except for coral). The areas of disinfection and sterilization (A61L) were particularly relevant for coral and sponges, therapeutics (A61P) was more pronounced in sponges, and the cosmetic area (A61Q) was particularly relevant for starfish. The food domain (A23L) displayed a more pronounced relevance in the context of molluscs and sea cucumbers. Peptides (C07K) showed higher relevance in all animal groups, except in sponges. In contrast, macromolecular compounds (C08H, C08L) and glue/gelatine preparations (C09H) were more relevant for sponges.

**Figure 8:**
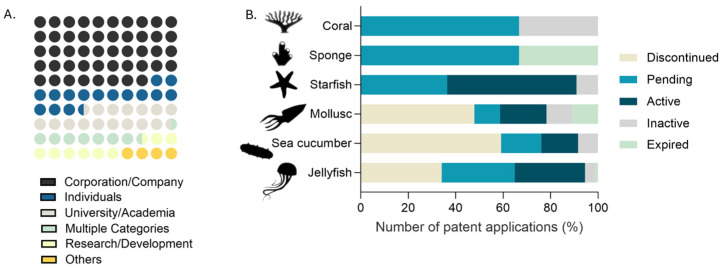
(**A**) Patents applicants by type of entity; (**B**) legal status of patents addressing collagens by taxonomic group of marine invertebrates (for simplification, please note that taxonomic groups are either phyla, in case of sponges and molluscs, or families within a phylum, in case of jellyfish, starfish and sea cucumbers).

The analysis of applicants per type of entities reveals that 48% of the patents are filed by either corporations or companies. This is followed by individual applicants and universities/academic institutions, each contributing around 16%. Research and development institutions account for 8% of patents, with a similar proportion being attributed to patents filed jointly by two or more entities. Concerning the legal status of the patents screened in the present study, 42% are classified as discontinued, with sea cucumbers and molluscs being the invertebrate groups with the highest number of contributions. A comparable number of patents are either pending (24%) or active (23%), while only a small portion is either expired (4%) or inactive (7%) (Figure 8).

### 2.3. Market Overview

Currently, bovine and porcine sources dominate the collagen market. However, globally, this trend is projected to decline due to concerns regarding disease transmission, possible allergic reactions and cultural impact. Beliefs, and other food and dietary restrictions and options, are also imposing a decrease in the sourcing of collagen from these more “conventional” raw materials. Indeed, society seems to be pushing to abandon the use of mammal-derived materials, and entrepreneurs in the biomedical arena report regulatory constraints on the use of mammal-derived tissues for collagen production (regarding the origin of such biological materials and associated safety issues). In contrast, marine collagens are considered to be safer, produced from more unobjectionable sources, being expected to experience the highest growth in consumption at the global level [91]. Additionally, the global production of marine collagens is relatively small compared to traditional bovine or porcine collagen [92] and, consequently, being even smaller for marine invertebrates, perhaps represents a niche market with high growth potential. The analysis of collagen market reports’ is usually focused on this market as a whole, encompassing collagens derived from various sources. Reports typically provide an overview of the whole collagen market, and, within this, marine collagens are often treated as one of the segmented categories; indeed, only a small number of reports specifically focus on the marine collagen market per se. According to these reports, the total collagen market was estimated to be valued at USD 5.1 billion in 2023 and is projected to grow to USD 7.4 billion by 2030, with a compound annual growth rate (CAGR) of 5.3% [93]. The Marine Collagen Market Research Report [94] summarizes trends in the marine collagen market per se, and this market is expected to grow from USD 2.79 billion in 2023 to USD 4.95 billion by 2032. The CAGR of this specific market segment is expected to be around 6.59% during the forecast period (2024–2032). Marine collagen is a small part of the collagen production volume but comprises almost half the revenue. This report suggests a shift towards personalized nutrition and targeted supplements designed to address specific health needs. Manufacturers are also exploring new sources of marine collagens, such as jellyfish, indicating as a justification the need to meet the growing demand for sustainable and ethical products [94]. While marine collagens sourced from fish are widely used in the food domain, as shown in the previous section, this report indicates that marine invertebrates are projected to experience the most rapid growth during the forecasted period, driven by increasing demand for marine collagens in the cosmetic and personal care sector. To illustrate this trend, Table 1 provides examples of products currently available on the market that incorporate collagens derived from marine invertebrates.

## 3. Discussion

### 3.1. Marine Invertebrate’s Taxonomic-Specific Insights on Research, Technological Advancements and Commercial Applications

In the 1960s, biochemical studies began addressing collagen phylogeny across marine phyla, from sponges to ascidians [37,105]. The search on this topic was further extended in the 2000s by genome sequencing [1]. More recently, three factors have renewed the interest in collagen: (1) the development (and price reduction) of -omics tools, improving phylogenetic studies; (2) the applications of collagen in the well-being sector; and (3) the use of by- and co-products in a sustainable and circular bioeconomy framework. The first factor, knowledge-driven, contributed to the emergence of comb-jellies, alongside sponges, as the earliest groups of marine invertebrates being addressed [8], suggesting that collagens from these organisms would be the ancestors of the animal structural proteins, including silks and keratins, with the former exhibiting features of these. The last two factors, application-driven, significantly boosted research and innovation on marine collagens, especially from marine finfish (from bony and cartilaginous organisms, such as sharks and rays) [10,106], particularly exploring extraction methodologies and industrial applicability, namely in human health and well-being. This boost in the search of marine collagens was also evident for marine invertebrates. As highlighted above, the scientific literature on collagens from marine invertebrates has experienced steady growth since the 2010s, with China and Japan being the main drivers of this trend, with cnidarians (e.g., jellyfish), echinoderms (e.g., sea cucumbers and sea urchins), molluscs (squids) and sponges supporting the development of applications targeting the use of these biomolecules in multiple biomaterials, in tissue engineering and exploring the antioxidant activity of collagen hydrolysates. This may be associated with the wider range of marine animals being explored in Asian countries when compared with Europe or North America, namely for food, with the availability of biomass triggering curiosity-driven research. Notably, China accounts for 22% of global publications in this area.

This emphasis is particularly relevant given the broader context of China’s scientific output, where 14.1% of its publications are in health sciences and 12.4% in biological and biomedical sciences, according to the National Science Foundation’s report on global scientific publications [106]. Compared to the United States, where invertebrate collagen papers identified in this study account for 7.1% of total publications and with the relevant publishing sectors according to NSF at 37.4 and 14.1%, respectively, the subject seems to be more heavily investigated in China. This broadening of marine sources for collagen may also be influenced from the sustainability demand side of the markets and consumers [107].

On the innovation and technological side, patent analysis revealed that among the taxonomic groups highlighted in the search performed in the present study, cnidarians and echinoderms stand out as the most significant marine invertebrate taxa in terms of innovation.

#### 3.1.1. Cnidarians

Among cnidarians, several species are being evaluated for their collagen content, primarily edible species from Asian countries and species that form blooms in the wild, such as those of jellyfish that are recurrently recorded in the Mediterranean [98,99]. In fact, the highest number of publications and patents within this group of invertebrates suggests a strong and global interest in the properties of collagen derived from jellyfish. Indeed, multiple industries, such as those from food, cosmetics and biomedicine, are interested in the presumably biological features displayed by collagen yielded from these marine invertebrates, namely its high biocompatibility, bioactivity, lower immunogenic and inflammatory responses, few biological toxins and/or contaminants [108,109]; additionally, the sustainability of sourcing some of these species from the wild, given the availability of its natural biomass and its composition as gelatinous organism, together with the know-how already in place to process collagen sourced from jellyfish, contributes to the growing interest displayed by multiple industries on this topic [110,111]. The co-word analysis supported this vision, as it revealed a cluster with the words “jellyfish”, “regenerative medicine”, “biomaterials” and “tissue engineering”, confirming that these topics are frequently studied together. In fact, recent works presented jellyfish collagen characterization studies for biomedical research, while others highlighted the interest of jellyfish decellularized matrices for the regeneration of different tissues or wound healing [50,99,112], just to name a few. Moreover, the examples of products found in the market concerning the use of jellyfish collagen also illustrate the economic potential of this type of collagen. Indeed, in Europe, the commercial exploitation of the bloom-forming jellyfish *Rhizostoma octopus* started in 2014 within UK waters, making it possible to extract high-quality collagen for biomedical applications [50].

#### 3.1.2. Echinoderms

Echinoderms are the second phyla with more research and patent activity in collagen from marine invertebrates. While part of this research is concerned with developmental biology, our co-word analysis revealed two distinct, yet complementary, areas of research within the Echinodermata. One cluster features sea urchins and their collagen, particularly regarding regenerative medicine and biomaterials, indicating ongoing studies into its biomedical applications but with no expression in the patent domain. In fact, research is studying tissues from the waste of the edible sea urchin Mediterranean–Atlantic species *Paracentrotus lividus*, as a source of high-value native collagen to develop innovative collagen-based biomaterials for tissue engineering as this collagen can be extracted without destructive methods, preserving its native fibrillar conformation and maintaining the structural and mechanical performance [85,88], contrasting with fish collagen that is produced as an acid-soluble protein, prone to thermal denaturation, thus resulting in gelatine. The second cluster of scientific publications focuses on sea cucumbers and starfish studies, with a particular focus on the structural and physicochemical properties of their collagen. These studies aim to understand how the unique characteristics of collagen yielding from these marine species influence the functionality and efficacy across a range of applications, including food, nutritional supplements, cosmetics, and biomedical products, in particular, that derived from edible species, such as the sea cucumbers *Stichopus japonicus* or *Apostichopus japonicus* [74]. The identification of mutable collagenous tissue in this class of organisms also motivated particular research efforts, mostly aiming to understand that dynamic phenomenon in which collagen seems to aggregate and disaggregate in response to external stimuli through the action of effectors and inhibition factors expressed by specialized cells [73]. Mimicking this behaviour in vitro would enable the establishment of smart materials with applications in different areas, but the light shed so far has not yet cleared the path. Noteworthy is the fact that patent analysis reveals that innovations related to sea cucumber collagen play a more significant role in the food domain than those from other taxonomic groups of marine invertebrates. Moreover, it also shows that these innovations are being developed mostly in China and the United States of America. China’s significant focus on sea cucumbers may be driven by its national culinary importance [113] but also by the growing demand for health-related products [114]. On the other hand, in the United States of America, the focus on sea cucumbers and starfish collagen research likely reflects the broader trend in the global market for alternative natural and marine-derived products, particularly for cosmetic and health applications [94].

While research and innovation on sea cucumbers have been ongoing for a longer period, both the scientific literature and patents concerning collagen derived from seastars indicate that both are recent and comparatively modest in relation to their jellyfish counterpart, possibly indicating an initial stage of collagen research for these groups. Furthermore, current research on starfish mostly focuses on species negatively impacting the shellfish farming industry and for whose biomass no feasible use has been found [81,115], suggesting a link between different blue bioeconomy industries in an effort to identify and use sustainable sources of collagen while simultaneously minimizing the ecological impacts of certain species.

#### 3.1.3. Sponges

Sponges have a relatively low number of patents filed to date. As shown by our analysis, with the exception of one publication in the 1970s, most scientific research on sponges addressing collagens has only gained momentum in the last two decades. Academic studies [12,29] and co-word analysis reveal an association between sponges and advancements in regenerative medicine, highlighting the growing interest in studying these organisms for biomedical research, not only due to the collagen contents in some species but also due to the peculiar morphological features or their bodies. Indeed, being porous to enable water flow, marine sponges’ “skeletons” resemble the porous templates used in tissue engineering for 3D culture of cells, which have already motivated the study of decellularized sponge biomass as natural scaffolds for regenerative medicine [116,117]. This trend suggests that sponges, as much as starfish, can also be considered an emerging group of marine invertebrates in the collagen landscape, even if sustainable exploitation is a critical concern. This interest may have been part of the drivers fostering sponge mariculture, whose discovery of bioactive metabolites and use for habitat restoration [118] has opened new routes for their valorisation. The case of the Mediterranean sponge *C. reniformis*, a sponge whose large-scale culture is being studied, is an example of how research is being paired with efforts to minimize environmental impact [32,34]. Other sponge species are also being investigated for their biomedical potential [24], though at present, there are no clear indications of sustainable exploitation methods for most of them. This gap points to the need for more research, focused not only on the biotechnological potential of sponges but also on developing environmentally responsible ways to harness their valuable properties.

#### 3.1.4. Molluscs

Mollusc collagen, particularly from squid, was also prominent in both the scientific literature and patent landscapes. In the scientific domain, molluscs appear in a cluster of studies that focus on the extraction and characterization of collagen, particularly from edible squid’s mantle and muscle tissues. Some of these studies were related to biochemical changes in the processing of squid tissues [119,120], while others were related to collagen isolation and bioactivity properties analysis [121,122]. Our findings suggest that research efforts are concentrated on understanding the biochemical and structural properties of squid collagen, as well as its functional derivatives, such as hydrolysates [123]. In terms of patents, molluscs, especially squids, are highlighted in food-related innovations. The extraction and use of squid collagen, for example, are frequently explored in patents aimed at developing new food products and bioactive peptides with functional health benefits, particularly in Asian markets [124].

### 3.2. Marine Invertebrate Collagen Market Trends—Challenges and Advances in Patenting

The majority of patents covering marine invertebrate collagen analysed in this study was linked to jellyfish or sea cucumber sourcing, clearly demonstrating the potential commercial value of these taxa. Additionally, it became clear that the types of commercial usages of these collagens vary with the marine invertebrate source—preparations for medical, dental or toiletry purposes were highly relevant across all sources (except for corals). On the other hand, therapeutics was more pronounced in sponges, and the cosmetic area was particularly relevant for starfish. In the food domain, molluscs and sea cucumbers had a more pronounced relevance. Interesting to notice was also that peptides and smaller molecules showed higher relevance across all analysed animal groups, except for sponges, whereas, in contrast, macromolecular compounds and glue/gelatine preparations were more relevant. These trends may indicate that some marine invertebrate collagen sources are better suited for certain types of applications, and future market developments can impact those populations unevenly. There is also a notable trend in the patent landscape concerning discontinued patents across the groups, especially related to mollusc collagen. These trends are observed for sea cucumbers as well. The high number of discontinued patents across these groups of marine invertebrates may suggest challenges in upscaling the processes and obtaining sufficient yields for these particular taxa. Additionally, downstream hurdles in patenting processes, regulatory hurdles, market demand or insufficient economic incentives to continue research in these areas are reasonable issues that may somehow justify these trends. Moreover, some patent applications are submitted as research project outputs and used as a tool to trigger new collaboration with companies to further explore such technological developments towards new products, but when such collaboration does not materialize or does not evolve as planned, the patent application is dropped. Nevertheless, a high percentage of active or pending patents, especially from jellyfish, suggests a strong ongoing innovation and potential commercial interest in collagen derived from this group of marine invertebrates. In fact, corporations and companies represent the majority of applicants, indicating a strong commercial interest in this source and form of collagen. These results are in line with the documented significant market potential and demand for collagen products, leading businesses to invest in research and patenting [94]. Universities and research institutions were also relevant applicants, which indicates an academic interest in technological transfer and innovation activities in this field and an alignment of their research and development efforts towards solving specific industry needs. Moreover, applicants falling into multiple categories also point to collaborative efforts among different entities, which may lead to more comprehensive approaches to collagen research, combining commercial insights with academic research [125].

### 3.3. Research Needs for Marine Invertebrate Collagens vs. Vertebrate Collagens

Recent research highlights differences in collagens between marine invertebrate and mammalian sources [50], which also affect their extraction methods. For instance, collagens from sea urchins and sponges can be extracted in their fibrillar conformation, preserving structural integrity. In contrast, collagens from vertebrates, such as fish, are typically produced as an acid-soluble protein, making them prone to thermal denaturation and often resulting in gelatine [76].

Regarding their properties, major differences between marine invertebrate collagens and mammalian collagens are reported, such as their amino acid composition and thermal stability (with terrestrial forms often exhibiting higher stability given the high content of the amino acid hydroxyproline) [10]. Also, variations in molecular weight between vertebrate and invertebrate forms [78,81,126] and different SDS-PAGE band patterns even within the same species [79,127,128] may suggest inconsistencies [50,78], and, therefore, traditional approaches for collagen molecular characterization are insufficient.

Proteomics offers deeper insights into collagen types and post-translational modifications (PTMs), often reported in collagen from marine invertebrates [23,58], which are crucial to the structure and function of collagen. Proteomics also enables the identification of co-factors that are part of the collagen matrix, providing insights into biological roles and the influence of environmental factors, both of which are relevant aspects to understand species- or taxa-specific collagen features [23,129]. A more detailed study of marine invertebrate collagen can also highlight specific and unique properties of such biomolecules compared to those of vertebrates or other marine taxonomic groups, adding further value to their potential applications and enhancing their commercial exploitation interest. For instance, collagen from jellyfish was reported to produce a low inflammatory response compared to the one of porcine origin [130]. Moreover, marine-derived collagen often presents fewer regulatory and quality control challenges [131]. However, as there is currently no high-throughput and low-cost method for determining collagen subtypes (mass spectrometry being a very common alternative), it seems likely that the most immediate research will still be dependent on the facilities within each laboratory working on this topic, and, thus, a large-scale subtype identification of these collagens will most likely remain unfeasible [132]. In another perspective, it is also necessary to better elucidate the performance variability, considering the health and well-being application, of collagen peptides, gelatine and native collagen. As native collagen is typically extracted via laborious processes involving acid extraction, or even more complex processes comprising matrix disaggregating solutions, with particular care with working temperature to avoid protein denaturation, it is generally considered simpler to produce gelatine by using hot water processing or extract collagen peptides directly via enzymatic hydrolysis, which, in general, are also less expensive processes [133].

### 3.4. Sustainability Considerations

As research delves deeper into the properties of marine invertebrate collagens towards their market applications, it becomes essential to also consider sustainability. While the emergence of greener solvents and processing methodologies will certainly bring positive contributions to the environmental impact of collagen extraction processes, sustainability assessment starts with the access to biomass. For example, sourcing collagens from some sharks and other cartilaginous fish species raises significant concerns due to the heavy pressures these species face. In contrast, utilizing coproducts from the fish-processing industry presents a more sustainable alternative for producing collagens [107]. Another pertinent example can be drawn from the jellyfish fisheries, initially pursued as an attempt to control invasive behaviours associated with certain bloom species that experience pronounced increases in biomass and have garnered attention for their potential as a sustainable source of collagen [111]. However, the complexities surrounding jellyfish populations and their ecological roles, and the downstream impact of these fisheries, underscore the importance of implementing ecosystem-based management strategies that consider the broader implications of jellyfish harvesting [134]. Another perspective falling under the topic of sustainability of jellyfish harvesting is the amount of water they contain and whether using resources to extract a compound or material that is 95% seawater [135] from the ocean is a sustainable approach (both environmentally and economically). For this approach to be economically sustainable, the price obtained from extracted collagen would have to be significantly higher when compared to other sources.

This knowledge could be beneficial for opening up new commercial opportunities within the market for marine invertebrate collagen exploitation.

## 4. Materials and Methods

### 4.1. Literature Review and Patent Data Extraction and Analysis

**Figure 9:**
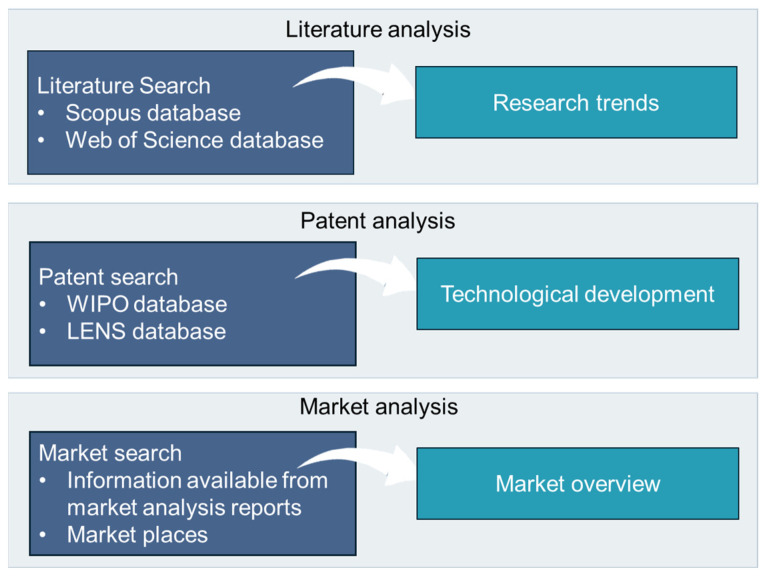
Databases and sources used for the scientific literature review, patent analysis, and market analysis to assess the significance of collagens from marine invertebrates in research, innovation, and market applications.

The present study evaluated the currently existing scientific literature and patents related to marine collagen derived from invertebrates. The search was performed across multiple databases (Scopus and Web of Science) in September 2024, following PRISMA guidelines [136], while patent-related information was retrieved from PATENTSCOPE (World Intellectual Property Organization) and the LENS database (Figure 9). The review process began with the identification of keywords related to marine invertebrate phyla described in the previous section. Twelve keywords were used to create a single query to each database. The keyword “collagen” was combined with marine invertebrates’ animals or groups: “marine invertebrate”, “marine sponge”, “coral”, “jellyfish”, “starfish”, “cucumber”, “shellfish”, “cuttlefish”, “squid”, “urchin” and “mollusc”. Boolean and proximity operators were used to refine the search. Table S1 includes all the combinations of keywords to retrieve the publications according to the requirements of each database.

A total of 691 publications were identified. After the removal of duplicates, the titles and abstracts of 424 publications were screened. Publications were considered eligible if they focused on marine invertebrates’ collagen and addressed at least one of the following topics: (i) molecular structure and synthesis of marine invertebrate collagen; (ii) collagen functionality; (iii) evolutionary aspects of collagen; (iv) collagen synthesis and degradation; (v) properties; or (vi) potential applications. Only articles written in English and with abstracts available were included, and, therefore, reviews, conference papers/proceedings and book chapters were excluded from this analysis.

After an initial screening, a final set of 323 publications was considered relevant to address the study objectives. The publications collected were analysed by country (based on the corresponding author’s affiliation), publication year and biomass source (information extracted from abstract reading). To obtain an overview of the applications covered in these publications, a bibliometric mapping approach was employed. Co-word analyses were performed using VOSviewer software (version 1.6.19, https://www.vosviewer.com/) to visualize the resulting networks.

For patent data, a similar approach was carried out. The initial patent count resulted in 501 patent families. After the removal of duplicates (i.e., patents that appeared more than once in the dataset and patents that belong to the same family), the titles and abstracts of 314 patent publications were screened. Patents were considered eligible if they focused on collagen from marine invertebrates and addressed at least one of the following topics: (i) extraction; (ii) processing; (iii) processing devices; or (iv) use of collagen for product development. After screening, a final set of 227 patent documents was selected for further analysis. These patents were then analysed based on the country of origin (determined by the location of the patent assignee), filing year and collagen source (information extracted from the abstracts). The publications and patent documents are listed in Tables S2 and S3. To analyse the application fields of the patent documents, the International Patent Classification (IPC) codes (https://www.wipo.int/classifications/ipc/en/, accessed on 2 September 2024), which are assigned to patents, were examined. These codes were utilized to categorize the technological areas associated with each patent and identify fields of application. To evaluate the type of organization using patents as a form of intellectual property protection in this field, the applicants’ information was organized by entity typology. The analysis also focused on the patent legal status.

### 4.2. Market Data Extraction and Analysis

To obtain an overview of the applications of collagen derived from marine invertebrates in the market, market research firms (Statistica, MarketsandMarkets, MordorIntelligence and MarketResearchFuture) were screened to search for free information available in their reports to obtain an overview of market trends and forecasts. The search was based on the keywords marine AND collagen, to narrow the search to marine sources only. Additionally, online marketplaces (e.g., Amazon) were also searched (on 4 November 2024) to identify products that currently use collagens from marine invertebrates. To extend to other specialized products, further searches were performed using Google to access company websites and product listings. All product data collected were then organized and analysed by product category (cosmetics, nutraceuticals, pharmaceuticals, food and beverage, biomedical) and animal source. This search aimed to gain insights into the current market landscape for (marine) collagen products and assess the range of their applications across different industries.

## 5. Conclusions

The geographical and temporal distribution of marine invertebrate collagen research publications and patents allows for a better understanding of how local marine biodiversity, economic priorities and strategies and sustainability frameworks shape scientific focus across the globe. Regions like China, with diverse fishery industries, lead in publications and patents. Commercial and technological interest is high in medical, dental, and toiletry applications, and emerging markets highlight cosmetics and personalized supplements as recent trends with increasing interest from various regions. Analysis of different marine invertebrate sourcing trends and matching commercial interests also pointed to clues on future impacts into certain taxa and their sustainability. Future research should focus on diversifying marine invertebrate sourcing but also optimizing extraction methods, including cascading approaches and circularity concepts, exploring novel applications, and addressing the ecological issues when considering exploiting an animal source. Emphasizing the unique physicochemical properties of marine invertebrate collagens, such as solubility and mechanical strength, seems to be crucial for their observed market interest and penetration, as these traits influence their performance in applications like tissue engineering and food products, which represent higher-valued markets. Tracking these challenges and trends in research and innovation can highlight global innovation hubs, emerging markets, and for new sustainable practices in the marine collagen industry.

## Figures and Tables

**Table 1:** Ingredients or products found in companies or marketplaces (verified on 4 November 2024), which advertise the incorporation of collagens derived from marine invertebrates. Note: Several marine collagen products in the market do not discriminate their source of origin, with several of them being simply presented as ocean collagen or fish/marine-derived collagen.

Brand/Company and Source		Country	Ingredient/Product	Application
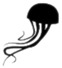	Jellagen [95]: Jellyfish	United Kingdom	Power (in pre-clinical stage)	Wound care
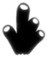	KliniPharm [96]: sponge	Germany	Boswellia Spongi^®^-Kollagen-Lecithin Capsules	Nutraceutical
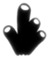	KliniPharm: sponge	Germany	Spongi Sunflower Collagen-Lecithin Capsules	Nutraceutical
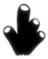	KliniPharm: sponge	Germany	SpongiCol^®^ Collagen-Lecithin Capsules	Nutraceutical
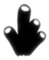	KliniPharm: sponge	Germany	SpongiCol Collagen-Lecithin Granulate	Nutraceutical
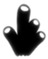	KliniPharm: sponge	Germany	SpongiCol ECO Collagen-Lecithin Capsules	Nutraceutical
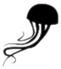	Certified Nutraceuticals [97]: Jellyfish	USA	kollaJell Brain Health & Cognitive function	Nutraceutical
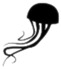	MitoLife [98]: Jellyfish	USA	Jellyfish Jolt (Wild-caught collagen)—capsules	Nutraceutical
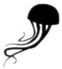	Neogenesis Health [99]: Jellyfish	South Africa	INFINITY JellyFish Collagencapsules	Nutraceutical
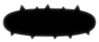	Nova Sea Atlantic [100]: Sea cucumber	Canada	Nova Sea Atlantic^®^ Sea Cucumber Capsules	Nutraceutical
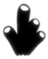	KliniPharm: sponge	Germany	ELEANA MARINE collagen creme nourishing sponge extract	Cosmetic
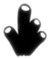	KliniPharm: sponge	Germany	ELEANA MARINE collagen serum reconstructing sponge extract	Cosmetic
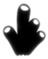	KliniPharm: sponge	Germany	ELEANA MARINE collagen gel nourishing sponge extract	Cosmetic
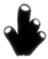	Nano Recipe [101]: sponge	South Korea	Korean Marin Collagen Concentrate Serum	Cosmetic
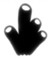	Joli Visage [102]	USA	Joli Collagen Spongilla Cream	Cosmetic
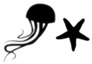	Coseed BioPharm [103] Jellyfish and starfish	South Korea	Jellyfish extract Starfish collagen	Cosmetic
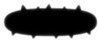	Bescher [104]: Sea cucumber	Australia	Sea cucumber collagen Anti-Ageing Cream	Cosmetic
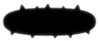	Bescher: Sea cucumber	Australia	Sea Cucumber Collagen Blemish Defence Serum	Cosmetic
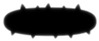	Bescher: Sea cucumber	Australia	Sea Cucumber Collagen Regeneration Serum	Cosmetic
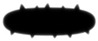	Bescher: Sea cucumber	Australia	Sea Cucumber Collagen Glow Toner	Cosmetic
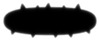	Bescher: Sea cucumber	Australia	Sea Cucumber Collagen Gel Cleanser	Cosmetic

## Data Availability

Data is contained within the manuscript and the Supplementary Materials.

## Supplementary Materials

The following supporting information can be downloaded at: https://www.mdpi.com/article/10.3390/md23010002/s1, Table S1: Query used to retrieve the scientific and patent publications. Table S2: Scientific publications selected for analyses. Table S3: Patent documents selected for analyses.
