# Supplementary material for: How Significant Are Marine Invertebrate Collagens? Exploring Trends in Research and Innovation
Source: Mar Drugs. 2024 Dec 24;23(1):2. doi: 10.3390/md23010002 (PMC11766948; doi:10.3390/md23010002)
Supplement: Supplementary file 1 [file marinedrugs-23-00002-s001.zip › Table S1.pdf]

Table S1. Queries used to search patent and scientific publications in Patent Scope, LENS, SCOPUS and WOS.

**Patent scope**
EN_TI: ((marine invertebrate NEAR3 collagen) OR (marine sponge NEAR3 collagen) OR (coral NEAR3 collagen) OR (jellyfish NEAR3 collagen) OR (starfish NEAR3 collagen) OR (cucumber NEAR3 collagen) OR (shellfish NEAR3 collagen) OR (cuttlefish NEAR3 collagen) OR (squid NEAR3 collagen) OR (urchin NEAR3 collagen) OR (mollusc NEAR3 collagen)) OR EN_AB: ((marine invertebrate NEAR3 collagen) OR (marine sponge NEAR3 collagen) OR (coral NEAR3 collagen) OR (jellyfish NEAR3 collagen) OR (starfish NEAR3 collagen) OR (cucumber NEAR3 collagen) OR (shellfish NEAR3 collagen) OR (cuttlefish NEAR3 collagen) OR (squid NEAR3 collagen) OR (urchin NEAR3 collagen) OR (mollusc NEAR3 collagen))
**LENS**
(title:("jellyfish collagen"~3) OR abstract:("jellyfish collagen"~3) OR claim:("jellyfish collagen"~3)) OR (title:("coral collagen"~3) OR abstract:("coral collagen"~3) OR claim:("coral collagen"~3)) OR (title:("cucumber collagen"~3) OR abstract:("cucumber collagen"~3) OR claim:("cucumber collagen"~3)) OR (title:("starfish collagen"~3) OR abstract:("starfish collagen"~3) OR claim:("starfish collagen"~3)) OR (title:("urchin collagen"~3) OR abstract:("urchin collagen"~3) OR claim:("urchin collagen"~3)) OR (title:("squid collagen"~3) OR abstract:("squid collagen"~3) OR claim:("squid collagen"~3)) OR (title:("shellfish collagen"~3) OR abstract:("shellfish collagen"~3) OR claim:("shellfish collagen"~3)) OR (title:("cuttlefish collagen"~3) OR abstract:("cuttlefish collagen"~3) OR claim:("cuttlefish collagen"~3)) OR (title:("marine sponge collagen"~3) OR abstract:("marine sponge collagen"~3) OR claim:("marine sponge collagen"~3)) OR (title:("mollusc collagen"~3) OR abstract:("mollusc collagen"~3) OR claim:("mollusc collagen"~3))
**Scopus**
TITLE-ABS-KEY (("collagen") W/3 ("marine invertebrate" OR "marine sponge" OR "coral" OR "jellyfish" OR "starfish" OR "cucumber" OR "urchin" OR "shellfish" OR "cuttlefish" OR "squid" OR "mollusc"))
**WOS**
("collagen") NEAR/3 ("marine invertebrate" OR "marine sponge" OR "coral" OR "jellyfish" OR "starfish" OR "cucumber" OR "urchin" OR "shellfish" OR "cuttlefish" OR "squid" OR "mollusc")
