# Supplementary material for: How Significant Are Marine Invertebrate Collagens? Exploring Trends in Research and Innovation
Source: Mar Drugs. 2024 Dec 24;23(1):2. doi: 10.3390/md23010002 (PMC11766948; doi:10.3390/md23010002)
Supplement: Supplementary file 1 [file marinedrugs-23-00002-s001.zip › Table S3.pdf]

Table S3. Patent documents selected for analyses.

Application Number	Title
CN 201710944013 A	A collagen anti-wrinkle facial mask and a preparing method thereof
CN 201710944014 A	A sea cucumber collagen extracting method
CN 201710944011 A	A sea cucumber collagen facial mask and a preparing method thereof
CN 201810351276 A	Anti-ageing and wrinkle-removing composition
CN 201810675745 A	Antibacterial hair conditioner capable of nourishing hair
CN 201610442962 A	Anti-infection medical film
CN 201610431320 A	Anti-infection surgical suture
CN 201610398184 A	Anti-inflammatory sterilizing adhesive for soft tissue
CN 201610998388 A	Anti-radiation capsule formula containing sea cucumber, lycopene and selenium and preparation technology of anti-radiation capsule formula
CN 201811333337 A	Anti-wrinkle essence liquid, preparation method and application thereof
CN 201610218531 A	Anti-wrinkle moisturizing EGF facial mask and preparation method thereof
CN 201610314067 A	Aquatic collagen peptide food used for adjuvant therapy of osteoporosis and arthritis
CN 202211047543 A	Astronavigation-level collagen peptide solid beverage and preparation method thereof
CN 201710544925 A	Beauty maintaining and young keeping sea cucumber cellulose fiber and making method thereof
CN 201610985159 A	Beer moisturizing water-replenishing essence liquid
CN 201510401806 A	Chlamydomonus nivalis cream and preparing method thereof
CN 201810041252 A	Collagen extracted from squid skin
CN 202322326049 U	Collagen extraction and separation device for cotton jellyfish
CN 201811019575 A	Collagen for skin repairing and preparation method of collagen
CN 202211518952 A	Collagen gel for injection without crosslinking agent residue and preparation method thereof
CN 202310989078 A	Collagen peptide liposome and moisturizing and anti-aging cosmetic
CN 201110439598 A	Collagen polypeptide drink and its preparation method
CN 201811567699 A	Collagen polypeptide regenerated cellulose fiber and preparation method thereof
CN 202322776114 U	Collagen powder canning bottle
CN 201811243231 A	Composite biological glue and preparation method thereof
CN 201910324477 A	Composition capable of replenishing essence, consolidating foundation, nourishing yin, moistening dryness, supporting yang and tonifying kidney and preparation method of composition
CN 201910192639 A	Composition for improving female microcirculation, regulating menstruation and warming uterus and preparation method thereof
CN 201910324536 A	Composition for nourishing yin, promoting blood circulation, tonifying kidney and resisting fatigue and preparation method of composition
CN 202311858975 A	Cuttlefish ink collagen jelly and preparation method thereof
CN 201821768045 U	Device for preparing sea cucumber collagen peptide by microbial fermentation method
CN 202310547004 A	Drug-loaded sea cucumber collagen-chitosan composite sponge particle as well as preparation method and application thereof
CN 202311234403 A	Enzymolysis preparation process of jellyfish collagen with natural structure
CN 201510044263 A	Essence containing chitosan oligosaccharide and jellyfish collagen and facial mask manufactured from essence
CN 202322328114 U	Extracting and separating device for collagen of orpilema esculentum
CN 202010200806 A	Extraction method of jellyfish collagen
CN 201710046259 A	Extraction method of sea cucumber collagen
CN 201811063213 A	Extraction method of sea cucumber collagen fiber
CN 200810013761 A	Extraction of jellyfish collagen and method for producing collagen peptide
CN 201410415356 A	Extraction process of jellyfish active collagen hydrolysate

CN 201510598815 A	Face-beautifying sea cucumber combination and preparation method thereof
CN 201410270094 A	Facial cleanser containing marine bioactive substances and preparation method thereof
CN 202310297408 A	Facial mask base cloth based on nano cellulose fibers and preparation method of facial mask base cloth
CN 202010358371 A	Food additive
CN 201510536663 A	Food processing technology of sea cucumber protein powder containing taurine and inositol
CN 202310743501 A	Formula of polypeptide honey beverage
CN 201711055902 A	Hand sanitizer applied to medical clinical laboratory and preparation method of hand sanitizer
CN 202111624398 A	Human body absorbable hemostatic sponge for promoting healing of diabetic wound and preparation method thereof
CN 201510537420 A	Instant sea cucumber preparation method
CN 202110885953 A	Jellyfish active protein as well as preparation method and application thereof
CN 200610042188 A	Jellyfish collagen and method for preparing the same
CN 202410113547 A	Jellyfish collagen mixture hydrogel composite functional dressing as well as preparation method and application thereof
CN 202321651663 U	Jellyfish collagen preparation device
CN 201610315809 A	Jellyfish collagen soap and preparation method thereof
CN 202310276222 A	Jellyfish collagen solution and nanofiber membrane for in-situ skin repair
CN 200710172710 A	Jellyfish derived kindó[illegible]collagen and preparation technique thereof
CN 202321622416 U	Jellyfish non-denatured collagen extraction device
CN 202311821792 A	Jellyfish, siraitia grosvenorii and codonopsis pilosula beverage and preparation method thereof
CN 200810150987 A	Limaigubang capsule
CN 201710849760 A	LMW (low molecular weight) jellyfish collagen peptide with activity as well as preparation method and application of LMW jellyfish collagen peptide
CN 201710508071 A	Making method for sea cucumber essence
CN 201811015005 A	Marine biological peptide acne-removing and repairing mask and preparation method thereof
CN 202110778937 A	Mask essence containing stem cell extracting solution and mask
CN 202111652787 A	Mask prepared based on jellyfish collagen solution and preparation method thereof
CN 201610439711 A	Medical film with function of swelling and pain relief
CN 201410691912 A	Medical hemostatic plugging dressing and preparation method thereof
CN 201910767476 A	Medicine formula for conditioning discomfort symptoms such as prostate frequent micturition and urgent urination
CN 202310035160 A	Method for continuously producing jellyfish collagen antioxidant peptide
CN 202311078854 A	Method for extracting collagen from jellyfish by low-temperature acid method
CN 201810086432 A	Method for extracting collagen from squid skin
CN 201110280516 A	Method for extracting collagen peptide from squid skin through chymotryptic hydrolysis
CN 202311766679 A	Method for extracting collagen peptide from starfish collagen and application
CN 200710159245 A	Method for extracting sea cucumber collagen and collagen
CN 201510464367 A	Method for extracting undenatured type II collagen from squid cartilage
CN 201110215798 A	Method for manufacturing collagen from black sea cucumbers from East China Sea
CN 200710013140 A	Method for preparing bioactive peptide of collagen from squid skin, and application
CN 201710552660 A	Method for preparing functional beverage through fermentation of jellyfish collagen peptide
CN 201810442260 A	Method for preparing functional beverage with taurine and black sea cucumber collagen oligopeptide
CN 201010144056 A	Method for preparing instant sea cucumber with stable collagen protein
CN 201310323629 A	Method for preparing medical collagen sponge from East sea dark gensing
CN 201510997484 A	Method for preparing medical wound dressing by using squid

CN 200810010618 A	Method for preparing sea cucumber collagen small peptide freeze drying powder extracted from sea cucumber decoction liquor
CN 201610870513 A	Method for producing high-water absorbing medical gauze
CN 201710794406 A	Mirex polluted sludge treatment agent and preparation method thereof
CN 201710676962 A	Moisturizing, anti-wrinkle and skin-whitening skin care product containing seaweed essence
CN 201510769412 A	Non-degenerated jellyfish collagen preparation method
CN 201910801507 A	Non-protein amino acid-modified collagen antioxidant peptide and preparation method thereof
CN 201910724594 A	Normal-temperature instant sea cucumber millet porridge and preparation method thereof
CN 202110611988 A	Normal-temperature preservation processing method of nutritional seasoning instant sea cucumbers
CN 202311078732 A	Novel method for determining jellyfish protein
CN 200710046306 A	Ocean cephalopod skin glue collagen and preparation method thereof
CN 200710171187 A	Oligopeptide collagen originated from cyanea nozakii and preparation technique thereof
CN 201611251111 A	Oxidation resistance enhancement-based making method of healthy and highly-nutritional food
CN 201610132686 A	Pectin and bacterial cellulose composite medical dressing and preparation method thereof
CN 202110605831 A	Peptidergic moisturizing essence and preparation method thereof
CN 201410684005 A	Polyurethane medical absorbable suture and preparation method thereof
CN 201110216444 A	Preparation method for decolorized squid skin collagen
CN 202311083630 A	Preparation method for extracting collagen in jellyfish through ethanol precipitation
CN 200810139279 A	Preparation method for holothurian collagen active peptide and application thereof
CN 201510148949 A	Preparation method for sea cucumber collagen beverage
CN 202211232102 A	Preparation method of collagen liquid
CN 201210013039 A	Preparation method of decolorized squid skin collagen
CN 202210053317 A	Preparation method of degradable antibacterial hemostatic hydrogel for promoting diabetes wound repair
CN 201810039592 A	Preparation method of Donghai black sea cucumber collagen antibacterial peptide
CN 201210416150 A	Preparation method of fresh jellyfish collagens
CN 201110427743 A	Preparation method of high-molecular-weight squid skin collagen
CN 201110246904 A	Preparation method of holozoic sea cucumber biological wine
CN 202310122780 A	Preparation method of jellyfish collagen active protein, composition capable of adjusting skin micro-ecology and membrane patch capable of improving skin inflammation
CN 201710451233 A	Preparation method of jellyfish collagen emulsion
CN 202311567355 A	Preparation method of jellyfish shoulder plate collagen
CN 202311464679 A	Preparation method of low-molecular-weight jellyfish collagen peptide-calcium chelate
CN 201210079032 A	Preparation method of medical squid-skin collagen oligopeptide gel
CN 201610950790 A	Preparation method of moisturizing beer essence
CN 201910801506 A	Preparation method of novel taurine modified antioxidation peptide
CN 201810675988 A	Preparation method of rich-nutrient tea oil hair conditioner with effects of relieving itching and removing dandruff
CN 200810013282 A	Preparation method of sea cucumber active polypeptide under ultrasonic extraction
CN 201610157959 A	Preparation method of sea cucumber collagen fiber gel
CN 201210173006 A	Preparation method of sea cucumber collagens
CN 201810045601 A	Preparation method of squid skin micromolecular collagen powder
CN 201210058876 A	Preparation method of xanthine oxidase inhibitor
CN 202311256249 A	Preparation process method of Mellis-sea cucumber collagen peptide freeze-dried powder
CN 201210418817 A	Preparation technology of high-transparency squid skin collagen
CN 201710568430 A	Preparing method of skin repairing soap containing jellyfish protein
CN 202010386306 A	Probiotic sea cucumber collagen composite peptide solid beverage

CN 202211013760 A	Process for extracting oligopeptide and polysaccharide from sea cucumber processing byproducts
CN 202410490003 A	Processing technology method of functional food Jianbao nutrition composite tablet capable of increasing bone mineral density
CN 201710576157 A	Production method of sea cucumber mucopolysaccharides
CN 201710597695 A	Production method of squid skin collagen peptide instant noodle sandwich biscuits capable of nourishing skin
CN 202210211318 A	Protein compound liquid with wrinkle removing function
CN 201710740817 A	Refirming additive and application thereof
CN 201811123786 A	Rehydration processing method for semi-dried sea cucumbers
CN 202310850199 A	Salty taste enhancing peptide screened from sea cucumber collagen and application thereof
CN 201710985344 A	Scar removal composition containing marine life extracts and preparation method thereof
CN 201910006288 A	Sea cucumber collagen compound preparation suitable for sub-health people
CN 202211608589 A	Sea cucumber collagen peptide compound composition as well as preparation method and application thereof
CN 202320056488 U	Sea cucumber collagen peptide preparation device with drying structure
CN 201711225379 A	Sea cucumber collagen peptide tabletted sweets
CN 201710871658 A	Sea cucumber nutrient health porridge
CN 201920599922 U	Sea cucumber oligopeptide ultrasonic enzymolysis equipment
CN 201610115897 A	Sea cucumber polysaccharide and sea cucumber collagen polypeptide combined preparation method
CN 202011541957 A	Sea cucumber soup paste as well as preparation method and application thereof
CN 201711423395 A	Sea cucumber toilet soap and production method thereof
CN 202310694182 A	Sea cucumber-sourced marine collagen acne-removing repair cream
CN 201410498974 A	Sealant for interior decoration
CN 202210211374 A	Self-repairing medical mask and preparation process thereof
CN 200710046307 A	Shellfish jacket membrane collagen and method for making same
CN 201510117285 A	Skin comprehensive anti-aging water condensation and preparation method thereof
CN 202011391732 A	Sleep-aiding beauty mask and preparation method thereof
CN 201911276987 A	Squid skin collagen and rose extract compound beverage and preparation method thereof
CN 201810064862 A	Squid skin collagen film forming liquid and preparation method and application thereof
CN 201210444275 A	Squid skin collagen used as moisturizing and beautifying preparation of cosmetics
CN 202310806080 A	Squid-derived polypeptide with anti-osteoarthritis activity and preparation method thereof
CN 202310001108 A	Starfish-sourced collagen as well as preparation method and application thereof
CN 202110119343 A	Sunflower receptacle preparation, preparation method and application thereof
CN 201610394267 A	Surgical suture capable of promoting wound healing
CN 201610143571 A	Technique for preparing squid skin collagen peptide through enzymic method
CN 202310758549 A	Temperature-responsive jellyfish collagen hydrogel as well as preparation method and application thereof
CN 201110025788 A	Traditional Chinese medicinal composition and preparation method for granules thereof
CN 201610314068 A	Universal type aquatic collagen peptide food for wound healing
EP 19888525 A	Composition for scalp and hair
EP 01909798 A	Method for isolating collagen from marine sponge and producing nanoparticulate collagen, and the use thereof
JP 2021017582 A	Cationic jellyfish collagen
JP 2011167665 A	Differentiation-inducing method of stem cell
JP 2004015758 A	Edible chewing gum and method for producing the same
JP 2009015059 A	Immune activity enhancer containing jellyfish collagen
JP 2002160735 A	Jellyfish collagen

JP 2002263407 A	Method and system for recovering collagen from jellyfish
JP 2006059720 A	Method for producing acetic acid extract of jellyfish and peptide derived from jellyfish collagen
JP 2005236302 A	Method for recovering collagen from jellyfish
JP 2006147425 A	Novel heptapeptide and prolyl endopeptidase inhibitor
JP 2009048595 A	Processes for producing blood glucose level elevation suppressor and starfish collagen peptide using starfish collagen peptide as effective ingredient
JP 2002244645 A	Skin lotion
JP 25727696 A	Solid material
JP 18217295 A	Succedanious skin made from ocean living things
KR 20120004536 A	A cosmetic composition for improving human skin wrinkles damaged by ultra-violet rays
KR 20200146672 A	A Jellyfish Pretreatment Method for Collagen Extraction and a Method of Extracting Jellyfish Collagen by Using the Same
KR 20120068353 A	A processing method of leached tea by using squid skin collagen peptide and pine niddles and persimmon leaves for anti-aging and anti-oxidant care and the leached tea thereof
KR 20190136043 A	An ecofriendly bio-plastic using a jellyfish and the ecofriendly bio-plastic
KR 20190093269 A	Biodegradable resin composition and menufacturing method thereof
KR 20070028840 A	Collagen extracted from outer skin of cuttlefish and cosmetic mask pack containing the same
KR 20120016328 A	Collagen from squid muscle skin and alaska pollock and production method thereof
KR 20220120796 A	Composite Comprising Sea Squirt Extract and Starfish-derived Collagen Peptide Elastic Ethosome Comprising Same and Cosmetic Composition Comprising Same
KR 20190102518 A	Composition for manufacturing of biodegradable container using grain and carboxymethylcellulose
1020130155574	Cosmetic composition comprising jellyfish collagen hydrolysate as active ingredient, for anti-oxidation, anti-inflammation, whitening and anti-wrinkle
KR 20200077702 A	Cosmetic composition containing starfish collagen and Viburnum erosum fruit extract for moisturizing and firming the skin
1020120004536	Cosmetic composition for improving an anti-wrinkling property on the skin which is damaged by uv rays
1020200077702	Cosmetic composition for improving skin moisturization and elasticity, containing starfish collagen and viburnum erosum fruit extract
KR 20130155574 A	Cosmetic compositions for anti-oxidation, anti-inflammation, whitening and anti-wrinkle comprising collagen hydrolysate of jellyfish as an active ingredient
1020070028840	Disease free collagen simply extracted from the outer skin of cuttlefish for replacing collagen extracted from cow or pig and a cosmetic mask pack containing the same, showing unchanged functionality
1020190136043	Environmentally friendly bioplastic manufacturing method using jellyfish and environmentally friendly bioplastic manufactured thereby
1020110054168	Extraction method of a collagen composition from the skin of squid, and whole bean curd containing thereof
KR 20120120347 A	Jellyfish collagen peptide mixture
KR 20150111553 A	Manufacturing method of tablets for antioxidant and physical structure care and the product thereof
KR 20110054168 A	Method for extracting collagen composition from squid's endothelium for improving calcium absorptance, and whole soybean curd containing the collagen composition
1020150111553	Method for manufacturing antioxidant muscularity enhancing functional food and antioxidant muscularity enhancing functional food manufactured thereby
KR 20200059674 A	Method for Obtaining Collagen Peptide from Starfish Elastic Liposome Comprising Collagen Peptide Derived from Starfish and Cosmetic Composition Comprising Same
1020070018222	Method for preparing a peptide with skin wrinkle inhibitory activity from collagen of cuttlefish through enzyme treatment and ultrasonic wave treatment
1020200031000	Method for preparing eco-friendly bioplastic using jellyfish and eco-friendly bioplastic thereby
1020120068353	Method for preparing leached tea having anti-aging and anti-oxidant effects by using squid skin collagen peptides, pine needles, and persimmon leaves, and leached tea prepared thereby

1020080092606	Method for preparing whitened cuttlefish collagen-derived peptide using sodium hydroxide and sonication
KR 20050047528 A	Process for preparing collagen from starfish
KR 20080092606 A	The making methode of whitened peptide from squid collagen
KR 20070018222 A	The method of preparing peptide which have activity of repressing skin wrinkles from cuttlefish collagen
RU 2012135262 A	Agent, having antitumour, anticoagulant, wound-healing, anti-inflammatory and antioxidant activity, capacity to inhibit collagenase and angiotensin converting enzyme, and method for production thereof
TW 111109424 A	Jellyfish collagen and Bacillus subtillis natto ferment that can relieve symptoms of degenerative osteoarthritis and its preparation method wherein the rough cartilage surface in knee joint tissue sections is smoothed, and the cartilage layer is thickened
TW 107204509 U	Skin care product containing water-soluble jellyfish collagen and water-soluble chitin fiber
US 81246109 A	Colloidal collagen burn wound dressing produced from jellyfish
US 201715439094 A	Hydrolyzed jellyfish collagen types I, II, and V and use thereof
08405979	Invertebrate type V telopeptide collagen, methods of making, and use thereof
US 201113107299 A	Method for making self-assembling, collagen-based material for corneal replacement
US 202117798681 A	Method for obtaining collagen peptide from starfish, elastic liposome comprising starfish-derived collagen peptide, and cosmetic composition comprising same
US 201716330862 A	Method for producing collagen hydrogels
US 202217833346 A	Method for producing collagen hydrogels
US 201715591301 A	Method of producing jellyfish collagen extract
US 201313944917 A	Method of regenerating connective tissue with a scaffold of coral-derived collagen
US 771601 A	Use of jellyfish collagen (type II) in the treatment of rheumatoid arthritis
RU 2014000497 W	Collagen from jellyfish and method for producing same
PCT/GB2022/050521	Collagenous extracts for use as a medicament
IL 2013050119 W	Composites comprising collagen extracted from sarcophyton sp. Coral
PCT/IL2009/000334	Coral-derived collagen and methods of farming same
GB 2021050856 W	Jellyfish collagen use
GB 2021051491 W	Jellyfish collagen use
KR 2021001090 W	Jellyfish pretreatment method for collagen extraction, and jellyfish collagen extraction method using same
IB 2015054324 W	Marine-sponge type iv collagen membranes its production and biomedical applications thereof
KR 2014001986 W	Method for isolating collagen from jellyfish by using radiation
EP 2014057966 W	Method for the production of collagen proteins derived from marine sponges and an organism able to produce said proteins
US 2021/0054208 W	Produce, isolate and/or extract collagen and/or gelatin from animal cell lines and/or tissue explants
US 2007/0063882 W	Self-assembling, collagen based material for corneal replacement
